# MK-8776, a novel chk1 kinase inhibitor, radiosensitizes p53-defective human tumor cells

**DOI:** 10.18632/oncotarget.12311

**Published:** 2016-09-28

**Authors:** Kathleen A. Bridges, Xingxing Chen, Huifeng Liu, Crosby Rock, Thomas A. Buchholz, Stuart D. Shumway, Heath D. Skinner, Raymond E. Meyn

**Affiliations:** ^1^ Department of Experimental Radiation Oncology, The University of Texas MD Anderson Cancer Center, Houston, TX, USA; ^2^ Department of Radiation Oncology, The University of Texas MD Anderson Cancer Center, Houston, TX, USA; ^3^ Merck Research Laboratories, Boston, MA, USA; ^4^ Present address: Department of Radiation Oncology, Fudan University Shanghai Cancer Center, Shanghai, China

**Keywords:** radiation, Chk1, p53-dependent, MK-8776, DNA damage

## Abstract

Radiotherapy is commonly used to treat a variety of solid tumors but improvements in the therapeutic ratio are sorely needed. The aim of this study was to assess the Chk1 kinase inhibitor, MK-8776, for its ability to radiosensitize human tumor cells. Cells derived from NSCLC and HNSCC cancers were tested for radiosensitization by MK-8776. The ability of MK-8776 to abrogate the radiation-induced G2 block was determined using flow cytometry. Effects on repair of radiation-induced DNA double strand breaks (DSBs) were determined on the basis of rad51, γ-H2AX and 53BP1 foci. Clonogenic survival analyses indicated that MK-8776 radiosensitized p53-defective tumor cells but not lines with wild-type p53. Abrogation of the G2 block was evident in both p53-defective cells and p53 wild-type lines indicating no correlation with radiosensitization. However, only p53-defective cells entered mitosis harboring unrepaired DSBs. MK-8776 appeared to inhibit repair of radiation-induced DSBs at early times after irradiation. A comparison of MK-8776 to the wee1 inhibitor, MK-1775, suggested both similarities and differences in their activities. In conclusion, MK-8776 radiosensitizes tumor cells by mechanisms that include abrogation of the G2 block and inhibition of DSB repair. Our findings support the clinical evaluation of MK-8776 in combination with radiation.

## INTRODUCTION

The combination of molecular targeted agents with radiation for the treatment of human cancer continues to be an area of active investigation [1–3]. An emerging strategy in this regard involves the development of small molecule inhibitors of protein kinases that control cell cycle checkpoints [4]. Radiation and many cancer chemotherapy drugs kill tumor cells by inducing DNA damage. Such damage triggers a network of multiprotein complexes that initially sense the DNA lesions and subsequently signal their repair and this process has been referred to as the DNA damage response (DDR) [5]. The DNA lesions induced by radiation include single strand breaks (SSBs) and double strand breaks (DSBs) and these breaks activate ataxia telangiectasia mutated (ATM) and ATM and Rad3 related (ATR) [6]. ATM and ATR then activate the checkpoint kinases Chk1 and Chk2 which block cell cycle progression at multiple steps in G1, S and G2 phases to allow time for repair of the DNA damage prior to entry into mitosis [7]. Although generally, ATM activates Chk2 and Chk1 is activated by ATR, there is crosstalk between these pathways [8]. DSBs are considered the primary lethal lesion induced by radiation and cells that enter mitosis harboring DSBs would die due to massive chromosome aberrations [9, 10]. Thus, the G2 block which is mediated by Chk1 becomes critical for controlling cell survival following irradiation.

The progression from late G2 into mitosis is stringently controlled by the cdc2/cyclin B complex. This complex is activated by dephosphorylation of tyrosine 15 (Tyr15) on cdc2 by the phosphatase cdc25c thereby allowing entry into mitosis [11]. The G2 checkpoint is initiated in response to DNA damage by two seemingly redundant mechanisms downstream of Chk1. In the first, cdc25c is phosphorylated by Chk1 leading to its ubiquitination and subsequent degradation [12], thus, preventing activation of the cdc2/cyclin B complex. In the second, Chk1 phosphorylates the wee1 kinase activating it and stabilizing its presence [13]. Wee1 subsequently phosphorylates cdc2 on Tyr15 thereby inactivating the cdc2/cyclin B complex [14]. Initiation of the G2 block by these processes following DNA damage is especially critical for p53-defective cells. Cells with wild-type p53 can arrest cell cycle progression in G1 allowing time for repair whereas p53-defective cells totally rely on the G2 block for survival [15]. Therefore, based on the understanding of the mechanisms responsible for the G2 block and its importance in governing cell survival in response to DNA damage, there has been a substantial interest in the development of small molecule inhibitors of the G2 checkpoint for the sensitization of tumor cells to DNA-damaging cancer therapeutics including radiation—especially for tumor cells harboring defective p53 function [16, 17].

Several inhibitors of Chk1 have been developed and examined either as single agents or in combination with chemotherapy drugs and radiation for cancer therapeutics [8, 18]. Some of these agents have been assessed in clinical trials and the status of these trials has been the subject of a recent review [19]. One of the first Chk1 inhibitors to be examined in pre-clinical investigations and ultimately in clinical trials was UCN-01. Wang et al. [20] showed several years ago that UCN-01 potently and preferentially radiosensitized p53-defective cancer cells by abrogating the G2 block. Since then other Chk1 inhibitors have been studied for their radiosensitizing properties. The Chk1 inhibitors SAR-02106 and AZD7762 have been shown in pre-clinical studies to radiosensitize p53-deficient tumor cells in culture and human tumor xenografts made using p53-defective cells [21, 22]. Two additional Chk1 inhibitors, XL-844 and PF-00477736, are also capable of radiosensitization [23, 24]. Finally, MK-8776 (previously known as SCH900776), a selective Chk1 inhibitor [25], has been shown to sensitize pancreatic cancer cells to gemcitabine and radiation [26].

The alternative strategy for abrogation of the G2 block as mentioned above involves inhibition of the wee1 kinase. Thus, small molecule inhibitors of wee1 have also been developed [27] and two such agents have been examined in pre-clinical studies, PD-166285 and MK-1775 [28]. The radiosensitizing effects of PD-166285 have been described [29] and we previously showed that MK-1775 radiosensitizes p53-defective cells using *in vitro* and *in vivo* models [30]. In the present report, we have investigated the radiosensitizing properties of the Chk1 inhibitor, MK-8776, on human non-small lung cancer (NSCLC) cells and cells derived from head and neck squamous cell carcinomas (HNSCC) and test the p53 dependency of the radiosensitization. We further report a comparison of the ability of MK-8776 and MK-1775 to radiosensitize these cell lines and, additionally, we examine whether combining MK-8776 and MK-1775 results in an additive radiosensitizing effect when compared to either agent alone.

## RESULTS

### MK-8776 radiosensitizes human tumor cells in a p53-dependent manner

Clonogenic survival curve assays were used to test the ability of MK-8776 to radiosensitize human tumor cells. Several cell lines were tested including human lines derived from NSCLC and HNSCC tumors. The p53 status of each of the lines that were used is known. In their original report on MK-8776, Guzi et al. [25] showed that concentrations of 125–250 nmol/L of MK-8776 were sufficient to inhibit Chk1's function. Thus, we used the concentration of 200 nmol/L in all further experiments and, for the survival curve assays, we used a treatment schedule of a 1 h pre-irradiation treatment followed by an additional 18 h of treatment after irradiation. We found that this concentration of MK-8776 and treatment schedule did not result in any appreciable cytotoxicity with drug alone thereby allowing maximum sensitivity for assessing radiosensitization. This treatment schedule was identical to that used in our prior study of the wee1 inhibitor, MK-1775 [30].

**Figure 1:**
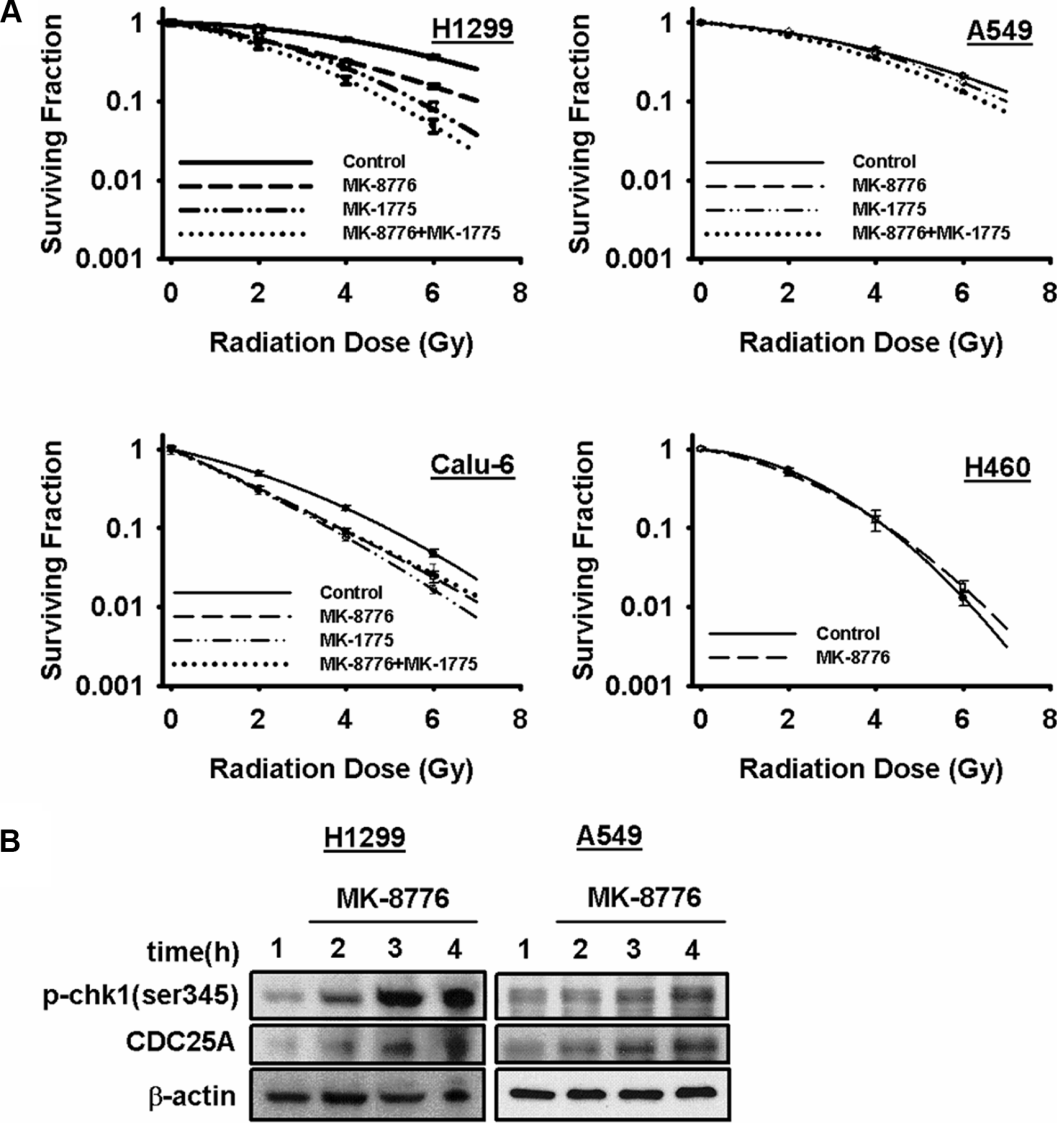
MK-8776 radiosensitizes NSCLC cells in a p53-dependent manner (**A**) clonogenic survival curves for A549 and H460 (both p53 wild-type) and H1299 and Calu-6 (both p53-defective) cells treated or not with 200 nmol/L of MK-8776 for 1 h prior to irradiation followed by an additional 18 h post-irradiation incubation in MK-8776 containing medium. The results shown represent the average of 3 or more independent determinations. Error bars are shown when larger than the symbol plotted and represent the standard error. (**B**) western blots for H1299 and A549 cells treated with MK-8776 for various times and assessed for expression of p-chk1 (ser345) and CDC25A.

Complete clonogenic survival curves for the 4 NSCLC lines examined consisting of two with wild-type p53, A549 and H460, and two that are null for p53, H1299 and Calu-6, were generated (Figure 1A). Lines with defective p53, H1299 and Calu-6, were significantly radiosensitized but lines with wild-type p53, A549 and H460, were not and this pattern extended to the p53-defective HNSCC line, FaDu (Supplementary Figure S1A). The degree of radiosensitization was quantified from the survival curves by comparing the surviving fractions at the radiation dose of 2 Gy (SF_2_) and by calculating the dose enhancement factor (DEF), i.e. the ratio of radiation doses to achieve a given survival level. The DEF values for all of the cell lines examined are provided in Table 1. SF_2_ is particularly relevant since 2 Gy is the typical dose given on a daily basis in clinical radiotherapy. All of the p53-defective cell lines had substantial and significant changes in SF_2_ values in response to MK-8776. For example, for H1299 cells, SF_2_ was reduced from 0.86 ± 0.02 in the control to 0.61 ± 0.02 (*p* < 0.05) by MK-8776 and for FaDu cells SF_2_ was reduced from 0.52 ± 0.07 in the control to 0.37 ± 0.04 (*p* < 0.05) by MK-8776. Based on the expectation that inhibition of Chk1 and wee1 might produce radiosensitizing effects by similar mechanisms, we compared MK-8776 and MK-1775 using survival curve analysis and assessed the combination of MK-8776 and MK-1775 for any additive effect. Four cell lines were used in this analysis, H1299, A549, Calu-6 and FaDu. The results, also shown in Figure 1 and Supplementary Figure S1, and quantified in Table 1 suggested that, in some of the p53-defective lines, wee1 inhibition by MK-1775 produced a slightly greater radiosensitization compared to Chk1 inhibition by MK-8776 but these differences were not statistically significant. Additionally, the combination of MK-8776 and MK-1775 appeared to radiosensitize some of the p53-defective cell lines to a slightly greater extent compared to MK-1775 alone but these differences were also not statistically significant. The p53 wild-type lines, A549 and H460, were not radiosensitized by any of these treatments including MK-1775 alone as we previously reported [30]. The normal lung fibroblast cell line, MRC-9, was also not radiosensitized by MK-8776 (Supplementary Figure S1).

Although the correlation shown in Table 1 between p53 status of a cell line and its radiosensitization by MK-8776 was evident for the panel of five tumor and one normal cell lines, we tested this relationship further using a cell line in which p53 expression is under exogenous control. Thus, we tested a cell line that we have reported on previously; H1299 cells that had been transfected with a Pon A-inducible p53 construct [31]. Immunoblot analysis (Supplementary Figure S1E) showed that this cell line did not express p53 when cultured in medium without Pon A but robustly expressed it when cultured for 24 h with Pon A. Clonogenic survival analysis of this cell line confirmed the p53 dependency of radiosensitization by MK-8776; radiosensitization was suppressed in these H1299 cells when p53 expression was induced by Pon A treatment (Supplementary Figure S1C) compared to the radiosensitization seen when Pon A treatment was withheld (Supplementary Figure S1D).

**Table 1:** DEF values for the cell lines used

Cell line	MK-8776	MK-1775	MK-8776 +MK-1775
	**p53 defective**		
**H1299**	1.53 ± 0.08*	1.73 ± 0.09	2.02 ± 0.10
**FaDu**	1.26 ± 0.07*	1.49 ± 0.21	1.55 ± 0.13
**Calu-6**	1.25 ± 0.06*	1.53 ± 0.03	1.40 ± 0.17
	**p53 wild-type**		
**A549**	1.00 ± 0.0	1.07 ± 0.05	1.16 ± 0.08
**H460**	1.03 ± 0.02		
**MRC-9**	1.00 ± 0.0		
	*Indicates *p* < 0.05		

MK-8776 at the concentration used was not appreciably toxic to any of the cell lines based on the plating efficiency of the unirradiated controls (Supplementary Figure S2). However, the combination of MK-8776 and MK-1775 appeared to produce a greater than additive cytotoxic effect in the p53 defective cell lines. Cdc25A is one of the main substrates for Chk1 and phosphorylation of Cdc25A by Chk1 targets it for ubiquitin-mediated proteolysis [12]. Thus, inhibition of Chk1 stabilizes Cdc25A protein levels. Furthermore, inhibitors of Chk1 interfere with the ATR/Chk1/PP2A feedback loop whereby Chk1 is continually phosphorylated by ATR on s317 and s345 and dephosphorylated by PP2A at these same sites [34]. Chk1 inhibitors, therefore, cause accumulation of s317 and s345. We used stabilization of Cdc25A and accumulation of s345 as markers for the ability of MK-8776 to inhibit Chk1 in H1299 and A549 cells (Figure 1B). The results indicated that MK-8776 inhibited Chk1 in both cell lines independently of their p53 status.

### MK-8776 abrogates the radiation-induced G2 block but to a smaller degree compared to MK-1775

**Figure 2:**
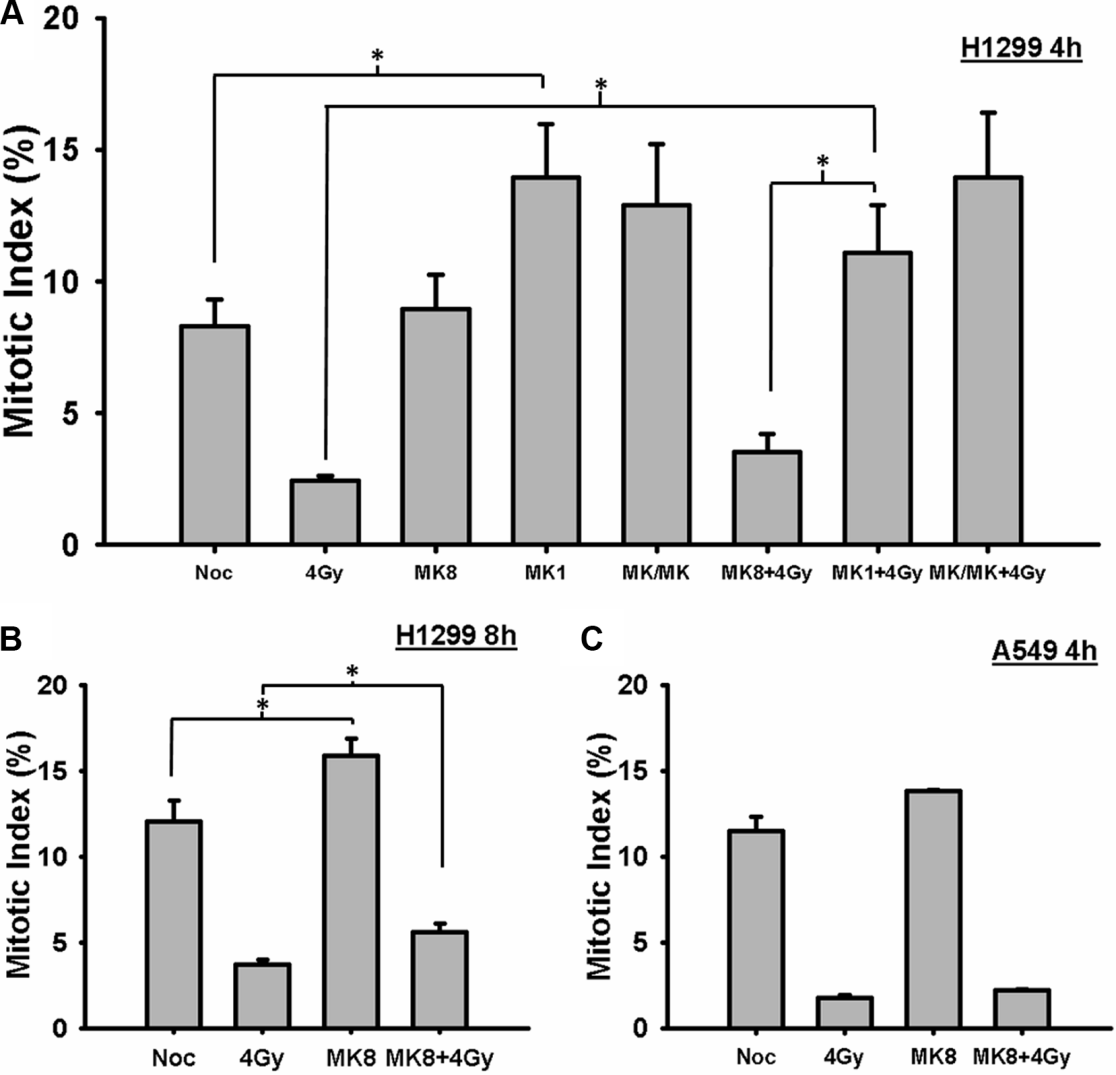
MK-8776 abrogates the radiation-induced G2 block (**A**) H1299 cells were irradiated with 4 Gy and the cells were then incubated in medium containing nocodazole for 4 h. Mk-8776 (200 nmol/L), MK-1775 (200 nmol/L) or their combination were added 1 h before irradiation. The entire cell population was harvested and analyzed for MI on the basis of p-HH3 as quantified by flow cytometry. (**B**) H1299 cells treated with MK-8776 similarly to A but harvested 8 h after irradiation. (**C**) A549 cells treated with MK-8776 similarly to A and harvested 4 h after irradiation. The values shown represent the average of 3 independent experiments. Error bars represent the standard error. ^*^ indicates *p* < 0.05.

We tested whether the radiosensitization effect of MK-8776 could be explained on the basis of an abrogation of the G2 block. In mitotic trap experiments. H1299 cells were treated with 200 nmol/L MK-8776 for 1 h, irradiated with 4 Gy, and then incubated for 4 or 8 h in medium containing nocodazole and MK-8776 and/or MK-1775. These samples were compared to control samples consisting of nocodazole alone, 4 Gy alone, MK-8776 alone, and to samples treated with MK-1775 and the combination of MK-8776 and MK-1775 with or without irradiation. All of the cells in the dishes were harvested at the end of the nocodazole treatment and the proportion of cells in mitosis (mitotic index, MI) was ascertained on the basis of p-HH3 stained cells as detected by flow cytometry. The results for the 4 h samples, depicted in Figure 2A, show that in H1299 cells MK-8776 alone did not accelerate un-irradiated cells into mitosis compared to the nocodazole alone control whereas MK-1775 treatment did replicating our previous finding [30]. Cells irradiated with 4 Gy displayed a reduced level of mitotic cells compared to the control consistent with a radiation-induced G2 block and the block was slightly reversed when the cells were treated with MK-8776 but this increase in MI compared to radiation alone did not reach significance. In contrast, MK-1775 abrogated the G2 block to a greater degree compared to MK-8776. The combination of the two agents was not significantly different from MK-1775 alone. In the 8-h samples (Figure 2B), there was evidence that MK-8776 accelerated unirradiated cells into mitosis and the radiation-induced G2 block was significantly reversed. A549 cells were assessed in a similar experiment (Figure 2C) using just MK-8776 where there was also no evidence of an abrogation of the G2 block at 4 h.

We also tested whether the radiosensitizing effects of MK-8776 correlated with abrogation of the radiation-induced G2/M block in asynchronously growing cells. A549 and H1299 cells were treated with 200 nmol/L MK-8776 for 1 h, irradiated with 7.5 Gy, returned to MK-8776-containing medium, and harvested at 4 h intervals for up to 16 h. Control cultures were irradiated but not incubated with the agent. Post-irradiation cell cycle kinetics were determined on the basis of G2/M-associated DNA content and MI on the basis of p-HH3 staining by flow cytometry. H1299 and A549 cells treated with radiation alone accumulated in G2/M over time peaking at 12 h after irradiation (Supplementary Figure S3), consistent with a radiation-induced G2 block. In both cell lines, following treatment with MK-8776 + radiation, the cells also accumulated in G2/M but to a somewhat lower level compared to the radiation control suggesting that MK-8776 has the ability to abrogate the G2-block and this effect becomes especially evident after 4 h. The results for the assessment of MI (Supplementary Figure S3) indicated that the ability of MK-8776 to accelerate irradiated cells into mitosis compared to radiation alone could not be detected in asynchronously growing cells and is best assessed using the mitotic trap approach as presented in Figure 2. To summarize the cell cycle progression results in Figure 2 and Supplementary Figure S3, it appears that abrogation of the G2 block by MK-8776 occurs to a similar extent in both H1299 and A549 cells, In addition, these results for MK-8776 markedly differ from those we reported previously for MK-1775 where this agent produced a substantial abrogation of the radiation-induced G2 block in the p53-defective H1299 cell line and robustly accelerated irradiated cells into mitosis [30]. Although it was assumed that MK-8776 and MK-1775 would induce radiosensitization by similar means, it appeared from these results that important differences between them exist.

### MK-8776 causes p53-defective cells to enter mitosis and into the next cell cycle harboring radiation-induced DSBs

**Figure 3:**
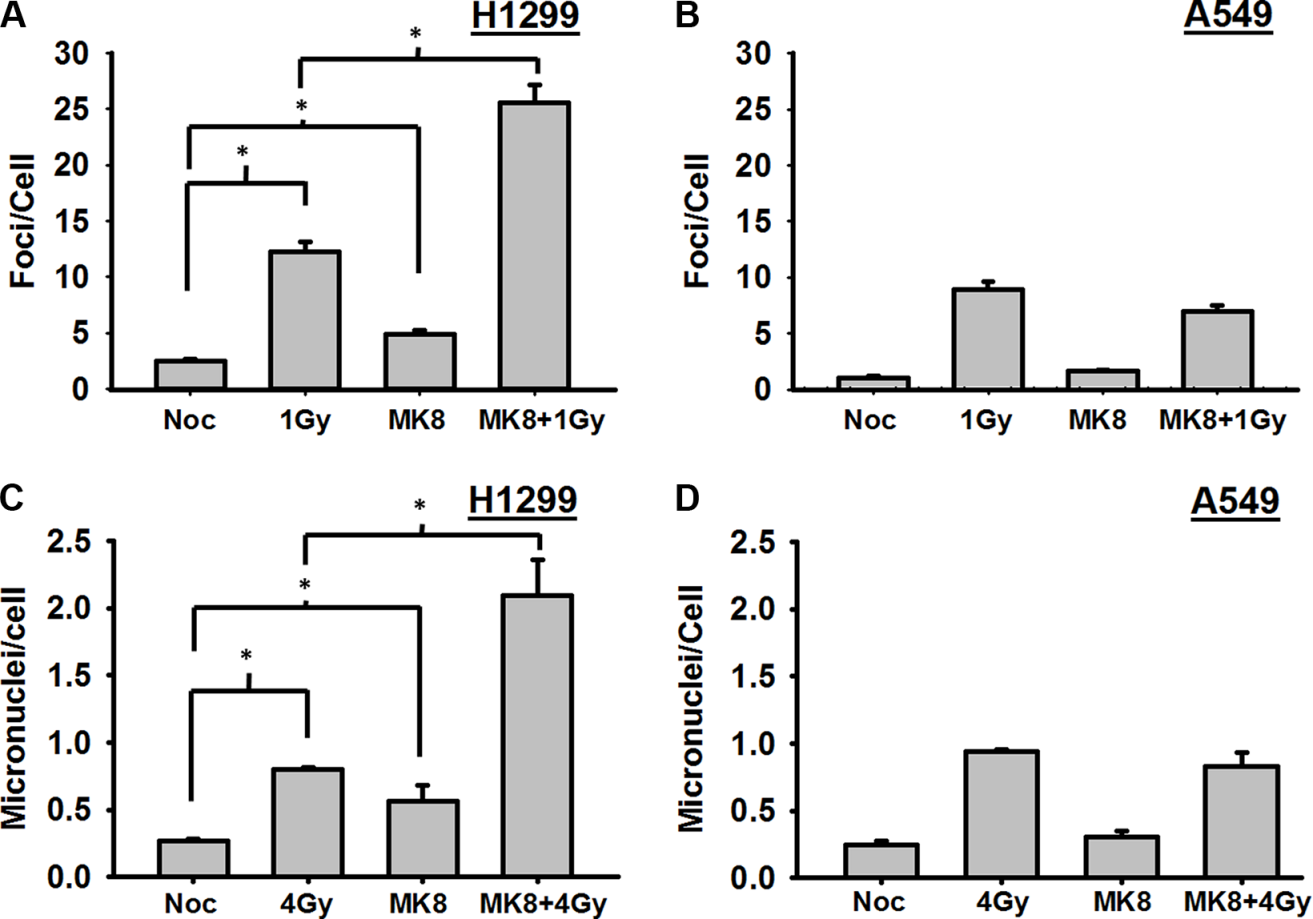
Cells prematurely accelerated into mitosis by MK-8776 harbor unrepaired DNA double strand breaks and undergo mitotic death (**A**) H1299 and (**B**) A549 cells growing on cover slips were irradiated with 1 Gy and then incublated in medium containing nocodazole for 4 h. MK-8776 (200 nmol/L) was added 1 h before irradiation. At the end of the 4 h nocodazole treatment, the mitotic cells (identified by their distinctive morphology) were analyzed for DSBs on the basis of γ-H2AX foci as detected by immunofluorescence. (**C**) H1299 and D, A549 cells growing in 100 mm dishes were treated as above except that the dose of radiation was 4 Gy. At the end of the 4 h nocodazole treatment, mitotic cells were harvested by gentle shaking and replated on cover slips in medium without nocodazole for 18 h. Cells were then harvested, stained with DAPI and analyzed for the presence of micronuclei. Error bars represent the standard error. ^*^ indicates *p* < 0.05.

As shown above, it appears that MK-8776 modestly accelerates irradiated cells into mitosis, and, thus, the radiosensitizing effect of MK-8776 could be explained if cells enter mitosis and progress into the next cell cycle before they completed repair of the radiation-induced DNA damage. In that case, unrepaired DSBs present at the time of mitosis would be expected to have lethal consequences. To test this, H1299 and A549 cells growing on cover slips were treated with MK-8776 for 1 h, irradiated with 1 Gy, and trapped in mitosis with nocodazole for 4 h. The mitotic cells in the samples were identified on the basis of their distinct morphology and γ-H2AX foci were scored in these mitotic cells by immunofluorescent staining as surrogates for radiation-induced DSBs. Mitotic H1299 cells that received a 1 h pre-irradiation treatment followed by continued incubation with MK-8776 harbored significantly more DSBs compared to radiation alone (*p* < 0.01) indicating that this agent allows irradiated cells to prematurely enter mitosis harboring un-repaired DSBs (Figure 3A). MK-8776 treatments did not similarly affect the levels of γ-H2AX foci in the A549 cells (Figure 3B). Representative photomicrographs illustrating the presence of γ-H2AX foci in H1299 cells following these different treatments are presented in Supplementary Figure S4A.

To test whether these un-repaired DSBs in mitotic cells would eventually be converted to lethal lesions, a similar experiment was conducted to assess the induction of micronuclei. Cells were grown in culture dishes, irradiated with 4 Gy, and, after the 4 h incubation in nocodazole, the mitotic cells were preferentially harvested by gentle shaking and replated onto cover slips in fresh medium without nocodazole or MK-8776. After 18 h of incubation, the cover slips were collected, stained with DAPI, and scored for micronuclei. In both H1299 and A549 cells, the incidence of micronuclei increased significantly with radiation alone compared to unirradiated control. Treatment of H1299 cells with MK-8776 led to substantially increased numbers of micronuclei compared to radiation alone (Figure 3C). In A549 cells, MK-8776 did not cause increased numbers of micronuclei over the radiation alone control (Figure 3D). Representative photomicrographs illustrating the presence of micronuclei in H1299 cells following these different treatments are presented in Supplementary Figure S4B. Taken together, the results for the DSB foci in mitotic cells and assessment of micronuclei (Figure 3) suggest that, although there may be some modest abrogation of the G2 block in both H1299 and A549 cells at later times after irradiation, the preferential ability of MK-8776 to radiosensitize p53-defective cells may be due to some suppression of DSB repair by MK-8776 in such cells at early times after irradiation that does not occur to the same extent in p53 wild-type cells.

### MK-8776 suppresses the repair of radiation-induced DSBs in a p53-dependent manner

**Figure 4:**
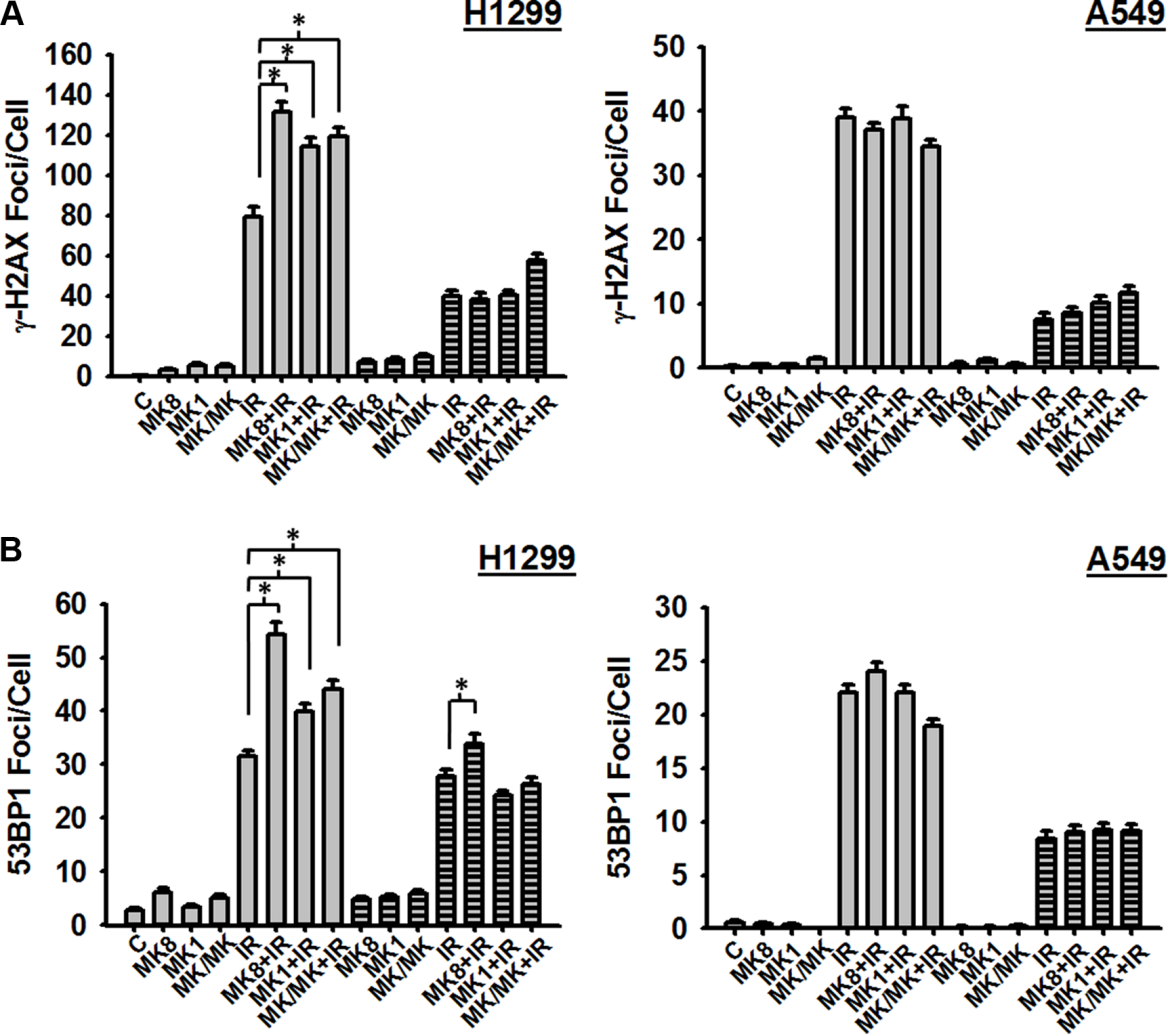
MK-8776 enhances the presence of radiation-induced DSBs in H1299 cells H1299 and A549 cells were treated or not with 200 nmol/L MK-8776 (MK8), 200 nmol/L MK-1775 (MK1), or their combination (MK/MK) for 1 h prior to irradiation (IR) with 1 Gy (**A**) or 2 Gy (**B**). Samples were then incubated for various times after irradiation and analyzed for DSBs on the basis of γ-H2AX foci (A) or 53BP1 foci (B) as detected by immunofluorescence. Agents were present during the post-irradiation incubations where indicated. A. Cells were harvested either 30 m (open bars) or 4 h (hatched bars) after irradiation. (B). Cells were harvested either 1 h (open bars) or 4 h (hatched bars) after irradiation. Each data point represents the average foci per cell from an analysis of 50 cells per sample. Error bars represent the standard error. ^*^ indicates *p* < 0.05.

The presence of unrepaired DSBs in mitotic cells following treatment with MK-8776 (Figure 3) suggested that this Chk1 inhibitor has a suppressive effect on the repair of these lesions possibly explaining its radiosensitizing effect. To test this possibility, we assessed the induction and repair of radiation-induced γ-H2AX foci as surrogates for DSBs. H1299 and A549 cells were treated with MK-8776, irradiated with 1 Gy, and cells were harvested at 30 m and 4 h after irradiation and analyzed for γ-H2AX foci. Treatments with MK-1775 were included in order to compare these 2 agents. The results (Figure 4A) indicate that in H1299 cells MK-8776 enhanced the presence of γ-H2AX foci at 30 m after irradiation compared to radiation alone. Similar effects were observed with MK-1775 and the combination of MK-8776 and MK-1775 although these treatments did not enhance foci to any greater extent than did MK-8776 alone. Foci levels at 4 h after irradiation indicated that substantial repair of DSBs had occurred and that neither MK-8776 nor MK-1775 when used as single agents had suppressed DSB repair over this 4 h time period. However, the combination of these 2 agents did appear to cause some suppression. Interestingly, both MK-8776 and MK-1775 appeared to induce γ-H2AX foci by themselves, especially at 5 h, consistent with reports in the literature that inhibition of either Chk1 or wee1 can induce DNA damage. In the case of A549 cells, when treated with the same protocol, MK-8776 did not appear to cause any enhancement of γ-H2AX foci compared to radiation alone and neither did MK-1775 at either time point.

To validate these results for γ-H2AX foci, we conducted a second set of experiments where we assessed radiation-induced DSBs using another known surrogate marker, 53BP1 foci. H1299 and A549 cells were treated with MK-8776, irradiated with 2 Gy, and cells were harvested at 1 h and 4 h after irradiation and assessed for 53BP1 foci. The results (Figure 4B) show that, similar to the results for γ-H2AX foci, in H1299 cells 53BP1 foci were enhanced at 1 h after irradiation following treatment with MK-8776 compared to radiation alone. This enhancement was not evident in the A549 cells treated in an identical manner.

In addition to Cdc25A, rad51 represents another important substrate of Chk1. Phosphorylation by Chk1 activates rad51 thereby coordinating its interaction with BRCA1 and facilitating HRR. Previous reports [21, 26] have indicated that Chk1 inhibitors including MK-8776 may inhibit HRR due to a suppressed activation of rad51 as detected on the basis of radiation-induced rad51 foci. We assessed the induction of rad51 foci in H1299 and A549 cells following treatment with MK-8776. The results (Supplementary Figure S5) indicated that MK-8776 modestly suppressed rad51 foci measured 4 h after irradiation with 4Gy compared to the radiation only control. However, this effect appeared to be independent of p53 status as it was essentially equal in both H1299 and A549 cells.

## DISCUSSION

In this study, we investigated the radiosensitizing abilities of a novel, selective inhibitor of Chk1, MK-8776. We focused our tests of MK-8776 on cell lines derived from types of human tumors, i.e. NSCLC and HNSCC, where radiotherapy typically plays a key role in the management of patients with these tumors. As shown in Figure 1 and Supplementary Figure S1 and summarized in Table 1, three p53-defective human tumor cell lines were radiosensitized by nanomolar concentrations of MK-8776 whereas two tumor cell lines with wild-type p53 and a cell line of normal tissue origin were not. However, to validate that radiosensitization by MK-8776 is p53-dependent, we also tested H1299 cells in which p53 expression had been restored using a Pon A-inducible vector. These results (Supplementary Figure S1C and D) confirmed the p53 dependence of MK-8776's radiosensitizing effect. We also compared the radiosensitizing effects of MK-8776 to the wee1 inhibitor MK-1775 and, finally, tested whether combining MK-8776 and MK-1775 produced an additive radiosensitization. A summary of these experiments indicates that MK-8776 and MK-1775 produce a similar degree of radiosensitization and that there is no additional sensitization with their combination. These findings suggested that these 2 agents radiosensitize via similar and/or overlapping mechanisms. However, additional studies revealed some important differences in their mechanisms of radiosensitization.

Numerous reports in the literature indicate that inhibition of either Chk1 or wee1 abrogates the G2 block and sensitizes cells to DNA damaging agents. We tested whether MK-8776 exerted such an effect in the context of its radiosensitizing effect. Using the mitotic trap approach, we did not observe any acceleration of irradiated H1299 cells into mitosis when treated with MK-8776 for 4 h after irradiation. This result was in stark contrast to MK-1775 where this agent accelerated both unirradiated and irradiated cells into mitosis prematurely as shown in Figure 2 and in our previous report [30]. The combination of MK-8776 and MK-1775 produced results similar to MK-1775 alone. Irradiated A549 cells were also not accelerated into mitosis by MK-8776 similar to the case for MK-1775 as we reported previously. In follow-up experiments using asynchronously growing cells, we did observe some abrogation of the G2 block at times exceeding 4 h after irradiation but the degree of abrogation was essentially identical in the p53-defective H1299 cells and the p53 wild-type A549 cells and was a much smaller effect to what we reported for MK-1775 in the H1299 cells [30]. Thus, in contrast to MK-1775, the preferential radiosensitization of p53-defective cells by MK-8776 could not be explained on the basis of an abrogation of the G2 block suggesting that MK-8776 and MK-1775 differ in their mechanism of radiosensitization.

MK-8776 and MK-1775 have both been previously reported to have effects on DDR processes [3, 4, 27]. Thus, we tested whether MK-8776 affected the presence of DSBs following irradiation. Although, as discussed above, MK-8776 did not accelerate irradiated cells into mitosis, we observed that H1299 cells that entered mitosis during the first 4 h after irradiation harbored substantial DSBs detected on the basis of γ-H2AX foci (Figure 3). A similar effect was not evident in the A549 cells. That these DSBs in H1299 cells contributed to radiosensitization was tested by assessing micronuclei in the subsequent cell cycle following harvest of the mitotic cells. Radiation-induced micronuclei are considered to be lethal lesions. H1299 cells which are radiosensitized by MK-8776 showed a substantial increase in micronuclei whereas A549 cells which are not radiosensitized by MK-8776 did not show any increase in micronuclei.

One interpretation of the presence of γ-H2AX foci in mitotic cells following the combination of radiation and MK-8776 as discussed above, is that MK-8776 suppresses the repair of radiation-induced DSBs, especially at early times after irradiation. To test this possibility, we assessed the induction of γ-H2AX foci as surrogates for DSBs at early times following irradiation. The results (Figure 4) indicated that MK-8776 enhanced γ-H2AX foci measured at 30 min after irradiation in H1299 cells but not in A549 cells. We interpret this as an inhibition of the fast phase of DSB rejoining by MK-8776. Previous assessments of the kinetics of DSB rejoining demonstrate a fast phase with half-times as short as 7 m and a slower phase that lasts for several h [35, 36]. No inhibition of DSB rejoining could be detected 4 h after irradiation. A similar effect was observed for H1299 cells treated with MK-1775 and the combination of MK-8776 and MK-1775 but neither of these treatments were substantially different from MK-8776 treatment alone generally correlating with the relative enhancements of radiosensitivity associated with these treatments (Figure 2 and Table 1). Measurements of DSB repair using γ-H2AX foci does not distinguish between the 2 major repair pathways relevant for ionizing radiation induced DSBs, homologous recombination repair (HRR) and non-homologous end joining (NHEJ) [3, 37]. Rad51 is a critical component of the HRR complex and, since it is known that Chk1 activates rad51 to facilitate its function in HRR [38], we assessed whether MK-8776 affected the formation of rad51 foci following irradiation. The results (Supplementary Figure S5) showed that, as has been reported previously for inhibitors of Chk1 [21, 26, 39], inhibition of Chk1 by MK-8776 suppressed the formation of rad51 foci at 4 h after irradiation to a small extent. However, since MK-8776 affected the formation of rad51 foci in both H1299 and A549 cells, this effect did not correlate with the findings for γ-H2AX foci (Figure 4) or the preferential radiosensitization of H1299 cells by MK-8776 (Figure 2). The protein, 53BP1, is a component of DDR that appears to facilitate the repair of DSBs via the NHEJ pathway by inhibiting critical steps in HRR [37] and assessment of 53BP1 foci is commonly used to specifically detect DSBs under repair by NHEJ. We measured 53BP1 foci at 1 and 4 h after irradiation in H1299 and A549 cells treated with MK-8776, MK-1775 and the combination. The results indicated that MK-8776 enhanced the presence of 53BP1 foci in H1299 cells at both the 1 h and 4 h time points but such an enhancement was not observed in A549 cells. Moreover, treatment of H1299 cells with MK-1775 produced a similar effect. Thus, it appears that the Chk1 inhibitor, MK-8776 radiosensitizes human tumor cells through an inhibition of NHEJ-mediated repair of radiation-induced DSBs and that occurs preferentially in p53-defective cells and that MK-1775 may have similar effects.

A broad interest has developed among cancer researchers in the preclinical assessment of checkpoint inhibitors as cancer therapeutics, especially inhibitors of Chk1 and wee1. The scope of what has already been accomplished in this area cannot be reviewed here but has been the subject of other reviews published recently [40–44]. It has been generally assumed that the antitumor activities of Chk1 and wee1 inhibitors are redundant because both types potently abrogate the G2 block through a shared pathway. However, it is now understood that, in addition to their regulation of entry into mitosis, Chk1 and wee1 both have important functions during S phase and can cause DNA damage including DSBs when inhibited. Following activation by ATR, Chk1 phosphorylates cdc25A which in turn induces the S phase checkpoint by reducing the cdk2/cyclin E complex leading to a slowing of DNA replication. Inhibition of Chk1, therefore, causes inappropriate DNA replication through collapse of stalled replication forks ultimately leading to DNA stand breakage [45, 46]. Inhibition of wee1, through increased cdk activity, stimulates DNA replication leading to nucleotide insufficiency. This, in turn, can lead to fork stalling followed by mus81 nuclease induced DNA strand breakage [47, 48]. To summarize, inhibition of either Chk1 or wee1 can cause DSBs in S phase [49]. Whether the mechanisms associated with the induction of these DSBs play any role in enhancing radiation-induced DSBs is not well understood. However, Chk1 has been reported to directly interact with DNA-PK, a critical component of NHEJ, thereby facilitating DNA-PK's end-joining activity [50]. Thus, it is possible that inhibition of Chk1 may suppress repair of radiation-induced DSBs through an abrogation of DNA-PK's important function in this pathway.

The anti-tumor activity of the combination of Chk1 and wee1 inhibitors has been tested in several previous reports [19, 51–55], generally indicating that the combination produces a synergistic effect on tumor cell proliferation or viability. We observed a similar effect when we examined the reductions in plating efficiency associated with these agents in our assessment of clonogenic cell survival (Supplementary Figure S2). These prior studies did not examine whether this combination enhanced tumor cell response to any anti-cancer therapeutic. The present report is the first to test this combination in the context of radiosensitization to the best of our knowledge. Thus, it appears that, although the combination of MK-8776 and MK-1775 may produce a greater-than-additive effect on cytotoxicity, there is no substantial enhancement of radiosensitization over that achieved by either agent alone. This lack of an enhancement by the combination suggests overlapping or redundant mechanisms of action for the 2 drugs. Our examination of mechanisms suggest that, although there are differences between them with regard to their ability to abrogate the G2 block or affect DNA repair pathways, it appears that both agents may radiosensitize p53-defective cells by prematurely accelerating them into mitosis before the radiation-induced DNA damage is fully repaired.

In conclusion, we have shown that the Chk1 kinase inhibitor, MK-8776, at nanomolar concentrations, potently radiosensitizes human tumor cells derived from NSCLC and HNSCC cancers in a p53-dependent manner. Similar to what we observed previously for the wee1 inhibitor, MK-1775 [30], the explanation for this sensitization appears to involve a drug-induced, premature acceleration of cells harboring unrepaired DNA lesions into mitosis leading to abnormal cell divisions and cell death. The results of the phase I trial of MK-8776 in combination with gemcitabine in patients with advanced solid tumors was recently published [56]. They report that the drug was well tolerated and indicate that some early evidence of clinical efficacy was observed. These clinical results coupled with the present report support the continued clinical assessment of MK-8776 in combination with DNA damaging agents including radiation.

## MATERIALS AND METHODS

### Cell cultures and reagents

The human cell lines A549 (ATCC Cat# CCL-185, RRID:CVCL_0023), H1299 (ATCC Cat# CRL-5803, RRID:CVCL_0060), Calu-6 (ATCC Cat# HTB-56, RRID:CVCL_0236), H460 (ATCC Cat# HTB-177, RRID:CVCL_0459), MRC-9 (ATCC Cat# CCL-212, RRID:CVCL_2629), and FaDu (ATCC Cat# HTB-43, RRID:CVCL_1218) were all obtained from the American Type Culture Collection (ATCC) and routinely maintained in RPMI-1640 medium supplemented with 10% fetal bovine serum (FBS), 10,000 U/ML of penicillin-streptomycin, and 2 mmol/L-glutamine. The identities of these cell lines were validated during this study by short tandem repeat (STR) profiling conducted by the institution's Characterized Cell Line Core using the AmpFlSTR Identifier PCR amplification kit (Applied Biosystems). The STR profiles for these cell lines matched their known ATCC fingerprints. The H1299 cells with ponesterone A (Pon A)-inducible p53 expression have been described previously [31] and were the kind gift of Dr. Jack Roth, Department of Thoracic Surgery, MD Anderson Cancer Center. MK-8776 and MK-1775 were provided by Merck Sharp & Dohme Corp., and their chemical structures have been described previously [25, 32]. Cells were trapped in mitosis using 0.2 μg/mL of nocodazole (Sigma-Aldrich).

### Antibodies

Antibodies to p-Chk1 (#2341), 53BP1 (#4937), β-actin (#4967L), and phospho-Histone H3 (p-HH3, #9706) were purchased from Cell Signaling Technology. Antibodies to cdc25a (sc-7389) and rad51 (sc-53428) were purchased from Santa Cruz Biotechnology, and γ-H2AX (Ser139) clone JBW301 (05–636) antibody was purchased from Millipore.

### Western blot analysis

Protein was extracted from the cell pellet using a lysis solution containing 50 mmol/L HEPES (pH 7.9), 0.4 mol/L NaCl, and 1 mmol/L EDTA. Phosphatase inhibitor cocktail 1 (10 μL/mL), 10 μL/mL phosphatase inhibitor cocktail 2, 10 μL/mL protease inhibitor (Sigma-Aldrich), and 1% NP-40 were added. Protein concentrations of the lysates were determined by the Bio-Rad protein assay. Equal amounts of protein were separated by 12% SDS-PAGE and transferred to an Immobilon membrane (Millipore). Protein bands were detected by incubating the membrane in primary antibody in 5% nonfat dry milk overnight at 4°C, followed by a 45-min incubation in the appropriate peroxidase-conjugated secondary antibody. The membrane was then developed with enhanced chemiluminescence with ECL plus Western Blotting Detection Reagents (Amersham) and visualized using film.

### Clonogenic assay

The radiosensitizing effects of MK-8776 were assessed by clonogenic assays. Briefly, cells growing in log phase were treated with 200 nmol/L MK-8776 1 h prior to irradiation. After irradiation, the cells were incubated for an additional 18-h post-irradiation treatment with 200 nmol/L MK-8776. The cells were then trypsinized and counted. Known numbers were seeded in 60-mm culture dishes in two sets of three for each dose of radiation. After 10–14 days, colonies were stained with 0.5% gentian violet in methanol and counted. The plating efficiency (PE) for each treatment was calculated by dividing the number of colonies by the number of cells plated and expressing the result as a percentage. The surviving fraction was calculated by dividing the PE for the treatment by the PE for the appropriate un-irradiated control.

### Cell cycle analysis

Cell cycle analysis was performed as previously described [30]. Briefly, cells were treated for 1 h with 200 nmol/L MK-8776, irradiated at 7.5 Gy, and then harvested at 0, 4, 8, 12, and 16 h later. The cells were then washed with PBS and fixed in 70% ethanol in PBS overnight at 4°C. The fixed cells were washed in Buffer A (0.5% bovine serum albumin (BSA) and 2% FBS in PBS) and then incubated in lysis buffer (0.1% Triton X-100, 0.5% BSA, and 2% FBS in PBS) on ice for 5 min. Cells were pelleted and then incubated with p-HH3 antibody at a dilution of 1:50 in Buffer A overnight at 4°C. The cells were then washed and incubated for 1 h in anti-mouse FITC secondary antibody at a dilution of 1:100. Cells were again washed, pelleted, and incubated in 2% BSA, 2% Tween-20, 5 mg/mL propidium iodide (PI, Sigma-Aldrich), and 2 mg/mL RNAse A (Sigma-Aldrich) for 1 h in the dark. Flow cytometric analysis was performed immediately thereafter using the institution's Flow Cytometry Core facility.

### Immunofluorescence

Immunofluorescence was performed as previously described [33]. Briefly, cells were cultivated on coverslips placed in 35-mm dishes and treated with radiation and/or drug as indicated. The medium was aspirated, and the cells were rinsed and then fixed with 2% paraformaldehyde for 15 m. Cells were permeabilized by a 10-m incubation with 100% methanol at −20°C. Following three 5-m rinses, the cells were incubated in blocking buffer (1X PBS, 50 μL/mL normal goat serum, and 0.3% Triton X-100) for 1 h at room temperature. Next, the cells were incubated in primary antibody in antibody dilution buffer (1X PBS, 10 mg/mL bovine serum albumin, 0.3% Triton X-100) overnight at 4°C with gentle shaking. Primary antibodies were visualized after a 2-h incubation with the appropriate Alexa Fluor-conjugated secondary antibody (goat anti-rabbit FITC or goat anti-mouse Alexa Fluor 594) at a 1:500 dilution. Nuclei were counterstained with 1:500 4′6-diamidino-2-phenylindole dihydrochloride (DAPI) in PBS. The coverslips were mounted on slides with Vectashield (Vector Laboratories) and analyzed using a Leica fluorescence microscope equipped with a CCD camera. Images were imported into Advanced Spot Image analysis software. To quantify repair foci, 50 nuclei were evaluated. Cells harboring micronuclei were identified by DAPI staining and quantified (200 cells/coverslip).

### Statistical analysis

Statistical significance was assessed by *t* test (two sample assuming unequal variances) and values are expressed as mean ± standard error. A difference was considered significant if *p* < 0.05.

## SUPPLEMENTARY MATERIALS FIGURES


