# Supplementary material for: MK-8776, a novel chk1 kinase inhibitor, radiosensitizes p53-defective human tumor cells
Source: Oncotarget. 2016 Sep 28;7(44):71660–72. doi: 10.18632/oncotarget.12311 (PMC5342109; doi:10.18632/oncotarget.12311)
Supplement: Supplementary file 1 [file oncotarget-07-71660-s001.pdf]

MK-8776, a novel chk1 kinase inhibitor, radiosensitizes p53-defective human tumor cells

Supplementary Materials

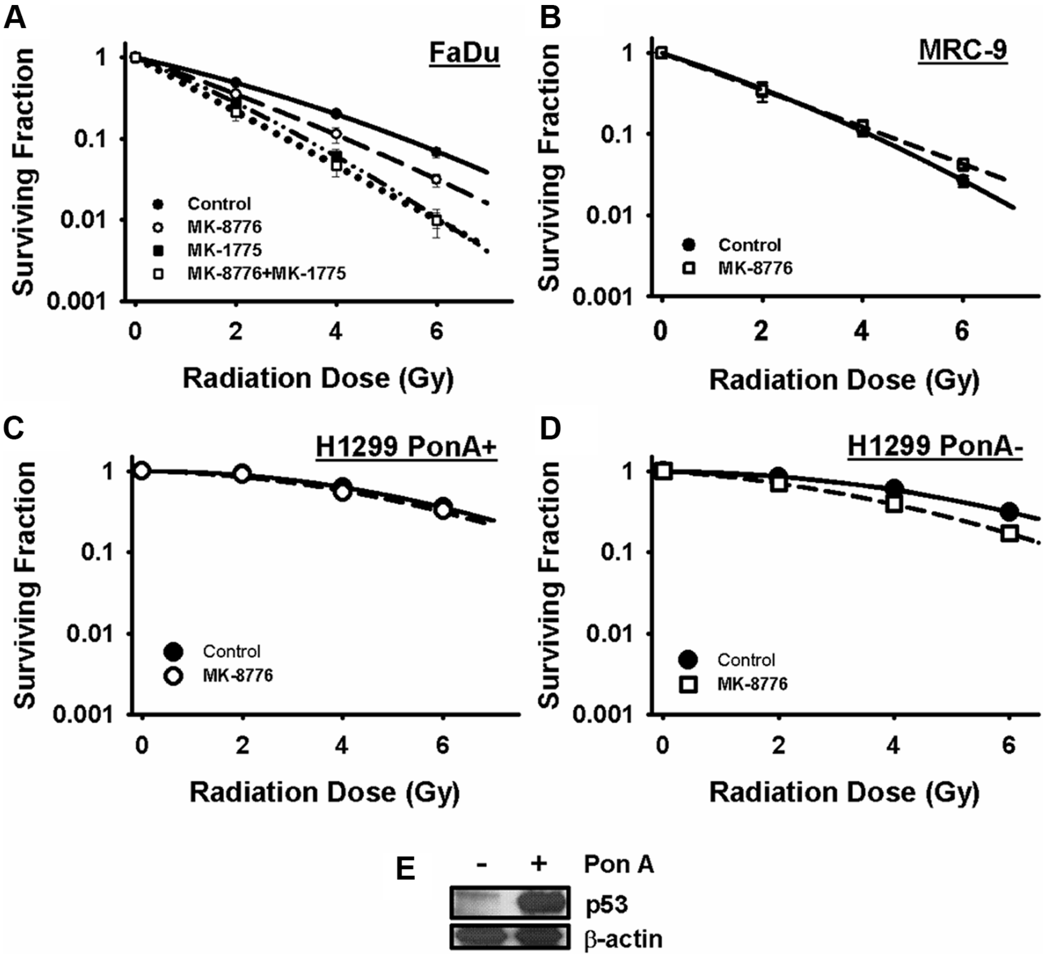

Supplementary Figure S1: MK-8776 radiosensitizes the HNSCC line, FaDu, (A) but not the normal lung fibroblast line, MRC-9 (B) as shown for clonogenic survival curves. Cells were treated or not with 200 nmol/L of MK-8776 for 1 h prior to irradiation followed by an additional 18 h post-irradiation incubation in MK-8776 containing medium. FaDu cells were also treated with MK-8776 or the combination of MK-8776 and MK-1775 similarly. Clonogenic survival curves for H1299 cells harboring a Pon A inducible p53 expression vector treated or not with MK-8776 as in panel A above, (**C** and **D**). Cells were treated with Pon A (5 μmol/L) for 24 h prior to irradiation (C) or not (D). The results shown represent the average of 3 or more independent determinations. Error bars are shown when larger than the symbol plotted and represent the standard error. (**E**) immunoblot showing p53 expression in H1299 cells harboring a Pon A inducible p53 expression vector. Cells were treated or not with Pon A for 24 h prior to harvest.

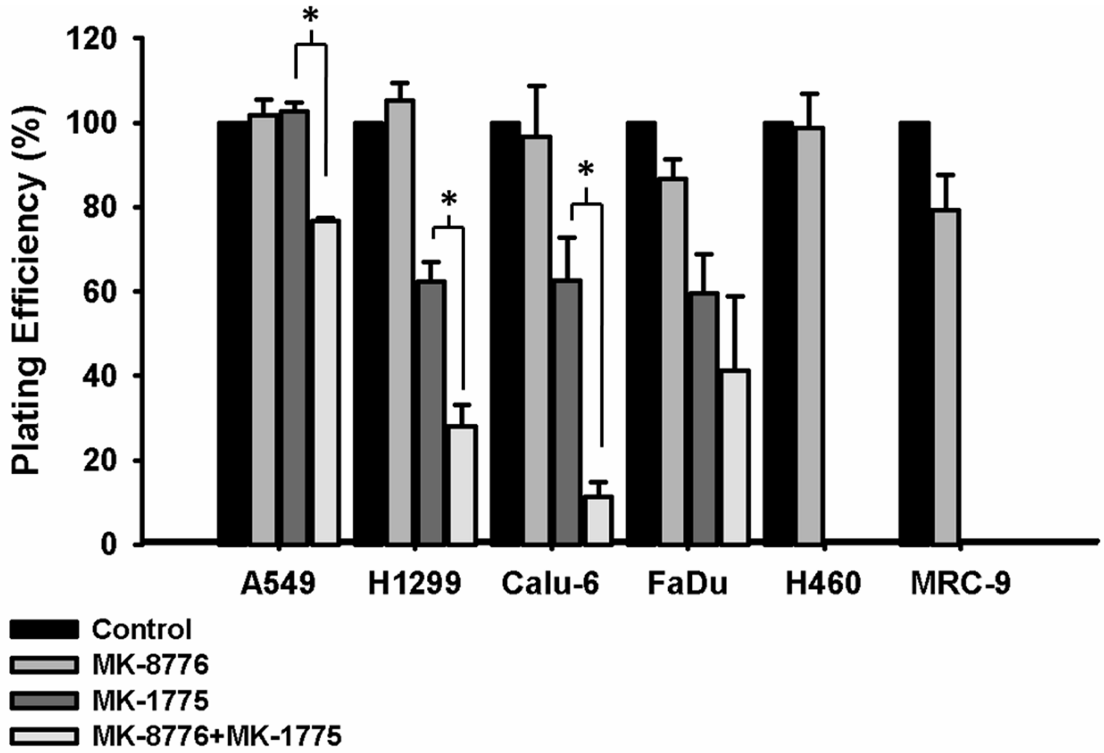

Supplementary Figure S2: Changes in plating efficiency for the cell lines used in this study following treatment with MK-8776, MK-1775 or the combination. The results are normalized to the untreated controls. The results shown represent the average of 3 or more independent determinations. Error bars are shown when larger than the symbol plotted and represent the standard error. * indicates $p < 0.05$.

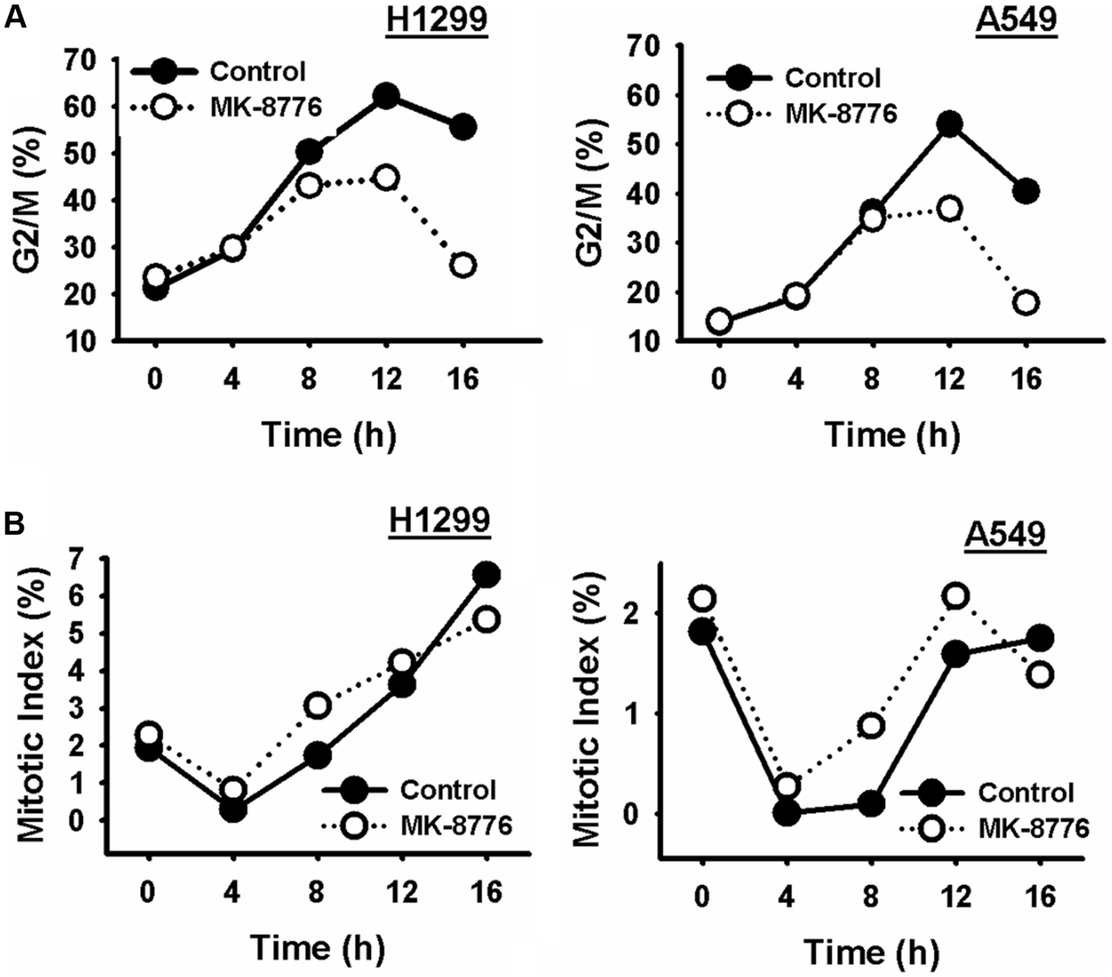

Supplementary Figure S3: Effects of MK-8776 on cell cycle kinetics following irradiation. Asynchronously growing H1299 and A549 cells were treated with MK-8776 for 1 h or not and then irradiated with 7.5 Gy. Samples were taken as a function of time thereafter and analyzed for percent G2/M (**A**) on the basis of DNA content and for mitosis (**B**) on the basis of p-HH3 by flow cytometry. Drug treatment was continued after irradiation in the MK-8776 treated cells. The data points shown represent the average of 2 independent experiments.

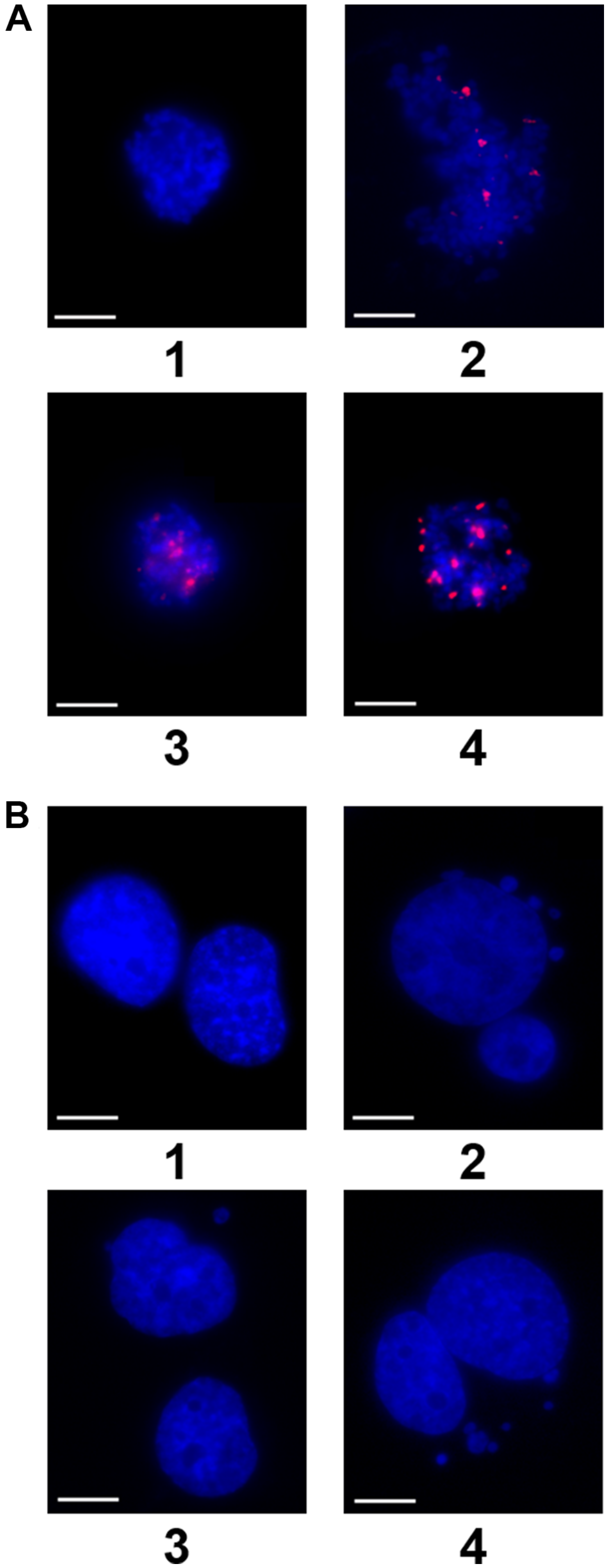

Supplementary Figure S4: Photomicrographs for Figure 3. (**A**) Representative photomicrographs illustrating the presence of γ-H2AX foci for the H1299 cells from the experiment depicted in Figure 3A. 1) nocodazole 4 h, 2) 1 Gy + nocodazole, 3) MK-8776 + nocodazole 4 h, 4) 1 Gy + MK-8776 + nocodazole 4 h, (**B**) Photomicrographs illustrating the presence of micronuclei for the H1299 cells from the experiment depicted in Figure 3B. 1) control, 2) 4 Gy alone, 3) MK-8776 alone, and 4) 4 Gy plus MK-8776. Bar is 10 microns.

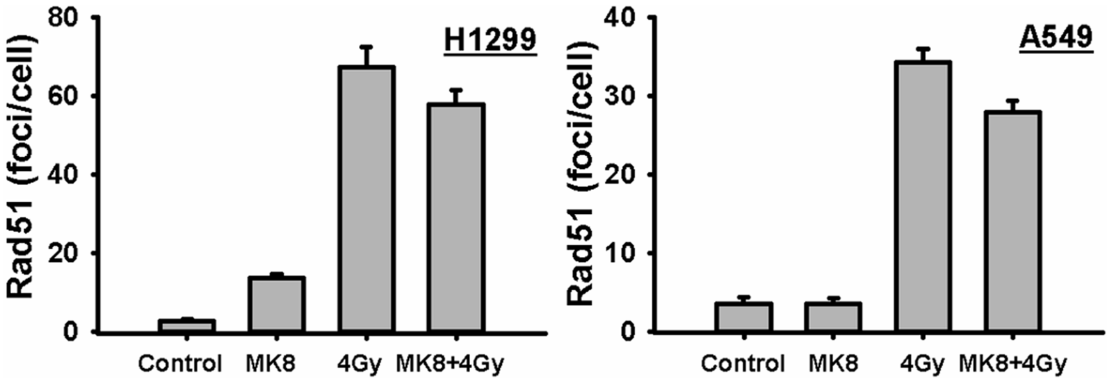

Supplementary Figure S5: MK-8776 suppresses the induction of radiation-induced Rad51 foci in H1299 and A549 cells. H1299 and A549 cells were treated or not with 200 nmol/L MK-8776 (MK8) for 1 h prior to irradiation with 4 Gy. Samples were then incubated for 4 h after irradiation and analyzed for DSBs on the basis of Rad51 foci.
